# Occurrence of gastrointestinal parasites in local ducks at varying altitudes in Yogyakarta, Indonesia

**DOI:** 10.14202/vetworld.2025.616-623

**Published:** 2025-03-18

**Authors:** Santika Anggrahini, Irkham Widiyono, Zein Ahmad Baihaqi, Ahmad Sofyan, Randi Mulianda, Wulandari Wulandari, Fitrine Ekawasti, Ima Fauziah, Sadarman Sadarman, Miarsono Sigit, Hendra Herdian, Riza Zainuddin Ahmad, Efi Rokana

**Affiliations:** 1 Research Center for Animal Husbandry, Research Organization for Agriculture and Food, National Research and Innovation Agency, Bogor, 16915, West Java, Indonesia; 2 Department of Internal Medicine, Faculty of Veterinary Medicine, Universitas Gadjah Mada, Yogyakarta, 55281, Indonesia; 3 Program of Animal Husbandry, Faculty of Agriculture, Universitas Islam Kadiri, Kediri, 64128, Indonesia; 4 Research Center for Veterinary Science, Research Organization for Health, National Research and Innovation Agency, Bogor, 16915, West Java, Indonesia; 5 Department of Animal Science, Faculty of Agriculture and Animal Science, Universitas Islam Negeri Sultan Syarif Kasim, Pekanbaru, 28293, Riau, Indonesia; 6 Department of Veterinary Reproduction, Faculty of Veterinary Medicine, University of Wijaya Kusuma Surabaya, East Java, 60225, Indonesia

**Keywords:** altitude variation, gastrointestinal parasite, local duck, nematode infection, seasonal occurrence

## Abstract

**Background and Aim:**

Gastrointestinal parasites are a common health concern in poultry, particularly in free-range farming systems. Local ducks (*Anas platyrhynchos javanicus*) in Indonesia are frequently exposed to various parasitic infections due to their extensive foraging behavior. Parasitic infections can reduce productivity, cause economic losses, and impact animal welfare. This study aimed to determine the occurrence of gastrointestinal parasites in ducks raised at different altitudes in Yogyakarta, Indonesia, and assess the influence of altitude and seasonal variation on infection rates.

**Materials and Methods:**

A total of 201 fecal samples were collected from local female ducks in three different altitude regions – coastal (<100 m), lowland (100–200 m), and highland (>200 m) – between November 2019 and May 2020, covering both the rainy and dry seasons. Fecal samples were manually collected and analyzed using the flotation method to detect parasitic eggs and oocytes. The occurrence of gastrointestinal parasites was statistically analyzed using Chi-square test.

**Results:**

Overall, 51.24% of the ducks were infected with gastrointestinal parasites. Nematodes were the predominant parasites, with *Capillaria* spp. (43.78%) and *Trichostrongylus* spp. (22.89%) being the most frequently detected species. Protozoa such as coccidia (5.97%) and cestodes like *Raillietina* spp. (1.49%) were also identified. The occurrence of infection varied significantly with altitude, being highest in lowland (61.43%) and highland areas (61.54%) compared to coastal regions (30.30%). Seasonal variation also influenced infection rates, with a higher occurrence observed during the rainy season (67.19%) than in the dry season (43.80%). Most infected ducks had single parasitic infections (80.39%), while mixed infections were less common.

**Conclusion:**

This study highlights the significant impact of altitude and season on the occurrence of gastrointestinal parasites in local ducks in Yogyakarta. The findings suggest that nematodes, particularly *Capillaria* spp., are the most persistent and widespread parasites. Implementing targeted deworming programs during the rainy season, improving farm sanitation, and educating farmers on parasite monitoring could help mitigate infections and improve duck health and productivity.

## INTRODUCTION

Ducks are a key source of animal protein in Indonesia. Ducks in Indonesia are generally raised extensively, where they are often released in the morning and evening, returning to backyard coops. Some ducks are also raised semi-intensively, housed in enclosures, and fed feed sourced from rice fields. Extensive farming systems are associated with low productivity and minimal inputs [1, 2]. Furthermore, regions with different topographies may experience varying rates of parasitic infection in poultry. Studies in tropical regions such as India [3], Ethiopia [4], and Bangladesh [5] provided comparable insights into parasite occurrence.

Poultry health issues are closely related to the incidence of helminthiasis [6]. Helminths of the gastrointestinal tract of poultry, classified as nematodes, cestodes, and trematodes, are dominated by nematodes, which significantly impact poultry due to their diversity and the intestinal damage they cause [7]. Various types of helminthiases can infect ducks, such as *Ascaridia galli*, *Heterakis gallinarum*, and *Capillaria* spp., with their occurrence varying based on environmental factors, farming systems, and farm management [8].

Temperature, humidity, and seasonality are important factors influencing the survival and epidemiology of parasites. Regions with low humidity tend to limit the survival of larvae in external environments [9]. In highland or lowland areas, soil tends to be more fertile, moist, and loose than in coastal areas, where soil is drier. Although parasites can adapt to temperature variations to develop into free-living stages, humidity remains a fundamental factor. Consequently, tropical regions with the highest rainfall are most favorable for the occurrence of gastrointestinal nematodes [10]. Given the regional variations in parasitic worm infections, monitoring these trends is essential for effective control strategies [11]. Therefore, this study aims to determine the occurrence of gastrointestinal parasites in ducks as influenced by altitude and season.

## MATERIALS AND METHODS

### Ethical approval

Ethical approval was obtained from the Faculty of Veterinary Medicine, Universitas Gadjah Mada, Yogyakarta, Indonesia (Approval No. 0013/EC-FKH/Int./2019).

### Study period and location

**Figure 1:**
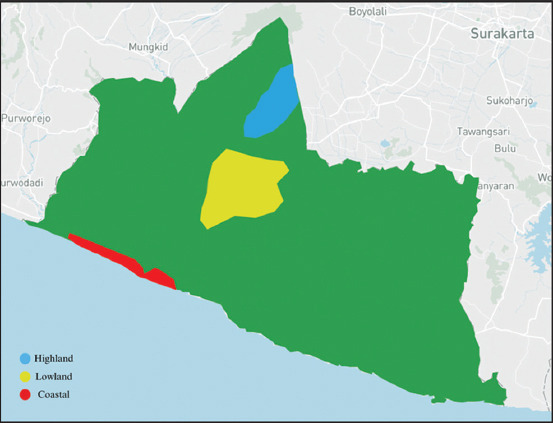
Geographical distribution of duck sampling regions based on altitude in Yogyakarta Province [Source: https://mapshaper.org].

The study was conducted from November 2019 to May 2020. Fecal samples were collected from local ducks raised in three regions at different altitudes in Yogyakarta Province. These regions include coastal areas at <100 m in the Galur and Temon sub-districts of Kulonprogo Regency, with average temperatures of 27°C–29°C and humidity levels of 75%–90%; lowland areas at altitude of 100–200 m across Bantul Regency and Yogyakarta City, with average temperatures of 25°C–27°C and humidity levels of 80%–90%; and highland areas at altitudes of >200 m in Cangkringan and Ngaglik sub-districts of Sleman Regency, with average temperatures of 23°C–26°C and humidity levels of 80%–90%. The above-mentioned regions are elaborated in Figure 1.

### Study design and sampling

The study design involved collecting one fecal sample for every ten ducks on the farm. Since most ducks raised in the local community are intended for egg production, the focus is primarily on rearing female ducks. Sampling was carried out during the rainy season (November 2019–December 2019) and the dry season (February 2020–May 2020). Fecal samples were collected from 201 local female ducks (*Anas platyrhynchos javanicus*), including 66 from coastal areas, 70 from lowland areas, and 65 from highland areas.

### Collection and examination of fecal samples

Fecal samples were randomly collected directly from individual ducks during morning and evening sessions, ensuring the upper layer of the feces was removed to avoid contamination. The analysis of these samples was conducted using the flotation method, which consisted of several steps: (1) One g of feces was placed in a plastic cup and mixed with 15 mL of saturated salt solution, then stirred with a mortar until homogeneous; (2) the homogeneous sample was filtered using a tea strainer, and the filtrate was transferred to a centrifuge tube up to a volume of 7.5 mL; (3) the centrifuge tube was balanced and centrifuged at 250× *g* for 5 min; (4) saturated salt solution was added until the liquid surface was slightly above the rim of the tube; (5) a cover slip was placed on the top of the tube and left for 5 min; and (6) finally, the slide was examined under a microscope (Olympus CX23 Japan), with the analysis conducted at the Veterinary Research Center Wates, Yogyakarta, and the Parasitology Laboratory of Universitas Gadjah Mada.

### Statistical analysis

The data obtained from the examination of gastrointestinal parasite occurrence were analyzed using descriptive statistics to determine infection rates across different altitude regions (coastal, lowland, and highland) and between seasons (rainy vs. dry).

The Chi-square test was employed to assess the statistical significance of differences in infection occurrence among the three altitude regions and between the two seasons. This test was used to determine whether parasite occurrence was significantly associated with environmental factors such as altitude and seasonality.

p < 0.05 was considered statistically significant. All statistical analyses were performed using SPSS software version 20.0 (IBM Corp., NY, USA). Data are presented as percentages and frequencies, with results summarized in tables and figures for better visualization.

## RESULTS

**Figure 2:**
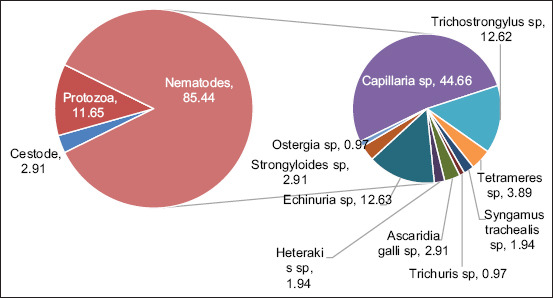
Occurrence of gastrointestinal parasites in all infected ducks(%).

Of the 201 local ducks examined, 103 (51.24%) were infected with gastrointestinal parasites. Various types of parasites were identified, with nematodes being the most common. *Capillaria* spp. had the highest occurrence, found in 46/103 (43.78%) infected ducks, followed by *Trichostrongylus* spp. which was detected in 13/103 (12.62%) infected ducks. In addition, protozoa, such as coccidia, were found in 12/103 (11.65%) infected ducks, while *Railletina* spp., from the Cestode group, was found in 3/103 (2.91%) infected ducks. Parasitic infections in infected ducks were predominantly caused by nematodes (85.44%), particularly *Capillaria* spp. (44.66%), followed by protozoa, specifically coccidia 11.65 (%) and Cestodes, represented by *Raillietina* spp. (2.91%) (Figure 2).

Table 1 illustrates the distribution of parasites in local ducks raised in coastal, lowland, and highland areas. Infection was detected in 20/66 ducks (30.30%) in coastal areas, 43/70 ducks (61.43%) in lowland areas, and 40/65 ducks (61.54%) in highland areas. Overall, the infection rate across the three locations was 51.24%. Among the 103 infected ducks, most had a single parasitic infection (82/103, 80.39%), while double infections were found in 18 ducks (17.65%) and triple infections in 4 ducks (3.92%) (Table 2). The distribution of nematode and protozoan parasites varied by altitude, as detailed in Table 3. *Capillaria* spp. was the dominant parasite in coastal areas, found in 11/66 ducks (16.67%). It was also the most common parasite in both lowland and highland areas, detected in 16/70 *(*22.85%) ducks and 19/65 (29.23%) ducks, respectively. In the lowlands, coccidia was the second most prevalent parasite, found in 11/70 (15.71%) ducks. This consistent pattern across different altitudes highlights the dominance of *Capillaria* spp. irrespective of geographical variation. Overall, nematodes, particularly *Capillaria* spp., were the most commonly found parasites in ducks across the three locations, with variations in occurrence depending on altitude.

**Table 1:** Gastrointestinal parasites infection in local ducks raised in different altitude regions.

Location	No. examined	No. positive	Positive (%)	Chi-square value	p-value
Coastal	66	20	30.30	2.919	0.039
Lowland	70	43	61.43		
Highland	65	40	61.54		
Total	201	103	51.24		

**Table 2:** Number of infections per parasite species in local ducks.

Location	No. positive	Single	Double	Triple	Chi-square value	p-value
Coastal	20	14	4	0	2.776	0.516
Lowland	43	33	8	4	2.764	0.017
Highland	40	35	6	0	2.777	0.010
Total (%)	103 (51.24)	82 (80.39)	18 (17.65)	4 (3.92)		

**Table 3:** Distribution and occurrence of gastrointestinal parasite infections in ducks.

Location	Parasite	No. infected	Occurrence (%)	Chi-square value	p-value
Coastal (n = 66)	Nematodes			2.44	0.086
	*Capillaria* spp.	11	16.67		
	*Ascaridia galli*	1	1.51		
	*Trichostrongylus* spp.	2	3.03		
	*Echinuria* spp.	3	4.54		
	*Tetrameres* spp.	1	1.51		
	*Heterakis* spp.	1	1.51		
	Protozoa				
	Coccidia	1	1.51		
Lowland (n = 70)	Nematodes			2.262	0.024
	*Capillaria* spp.	16	22.85		
	*Ascaridia galli*	2	2.85		
	*Trichostrongylus* spp.	3	4.28		
	*Trichuris* spp.	1	1.42		
	*Tetrameres* spp.	2	2.85		
	*Syngamus trachealis*	2	2.85		
	*Strongyloides* spp.	3	4.28		
	*Ostergia* spp.	1	1.42		
	Cestoda				
	*Railletina* spp.	2	2.85		
	Protozoa		15.71		
	Coccidia	11	15.71		
Highland (n = 65)	Nematodes			2.570	0.073
	*Capillaria* spp.	19	29.23		

**Figure 3:**
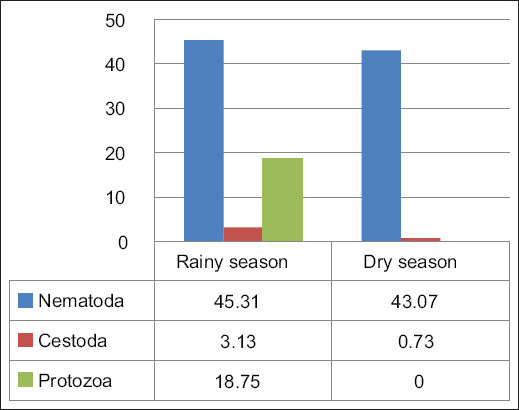
Occurrence of gastrointestinal parasites during the rainy and dry seasons (%).

The results presented in Table 4 indicate significant seasonal variation in the occurrence of parasitic infections in ducks, with a marked difference between the rainy season (November–December) and the dry season (February–May). The overall occurrence of parasitic infections was higher during the rainy season, detected in 43/64 (67.19%) ducks, compared to the dry season, where 60/137 (43.80%) ducks were infected (Figure 3). Understanding these seasonal dynamics is crucial for developing effective management strategies in duck farming and addressing public health concerns related to zoonotic infections.

**Table 4:** Overall occurrence of gastrointestinal parasites.

Parasites	No. infected birds	Infected birds (%)	Examined ducks (%)	Chi-square value	p-value
Nematodes	88	85.44	43.78	1.782	0.056
*Capillaria* spp.	46	44.66	22.89		
*Trichostrongylus* spp.	13	12.62	6.47		
*Tetrameres* spp.	4	3.88	1.99		
*Syngamus trachealis*	2	1.94	1.00		
*Trichuris* spp.	1	0.97	0.50		
*Ascaridia galli*	3	2.91	1.49		
*Heterakis* spp.	2	1.94	1.00		
*Echinuria* spp.	13	12.62	6.47		
*Strongyloides* spp.	3	2.91	1.49		
*Ostertagia* spp.	1	0.97	0.50		
Cestode	3	2.91	1.49		
*Railletina* spp.	3	2.91	1.49		
Protozoa	12	11.65	5.97		
Coccidia	12	11.65	5.97		
Total	103	100	51.24		

## DISCUSSION

### Occurrence of gastrointestinal parasites in ducks reared at different altitudes

Table 4 shows that *Capillaria* spp. (43.78 %) and *Trichostrongylus* spp. (22.89 %) are the most common nematodes. This finding aligns with previous research showing that nematodes are dominant parasites in poultry, with studies in Indiaand China similarly reporting a high prevalence of nematodes in ducks and other wild birds [12, 13]. Table 1 indicates that the occurrence of infection in lowland and highland areas is similar (61.43% and 61.54%), while coastal areas had a lower occurrence (30.30%). High humidity in lowland and low temperature in mountainous regions support the life cycle of parasites, particularly nematodes. Previous studies have found a higher prevalence in areas with elevated temperatures, although low temperatures are known to enhance egg and larval survival and increase infection rates, while the soil serves as an important reservoir and transmission site for the external larval stages of helminths and insects [14, 15]. In coastal areas, temperature and humidity tend to fluctuate and are relatively dry. According to Wang *et al*. [9], stable soil temperatures and adequate humidity significantly contribute to the survival of nematode larvae.

The majority of ducks experienced single infections (80.39%), whereas dual infections were found in 17.65% of ducks (Table 2). Mixed infections (involving two or more species of parasites) are often associated with more serious health impacts, as reported in similar studies on chickens in South Africa [16]. The occurrence of protozoan infections, specifically coccidia, was detected in 12/201 (5.97%) examined ducks (Table 4). Protozoa often emerge under poor hygiene conditions, particularly in free-range farms. A study in India also reported coccidia as a common protozoan in poultry with *Eimeria* species being highly prevalent, particularly *E. tenella*, *E. maxima*, *E. acervulina*, *E. mitis*, *E. necatrix*, and *E. brunetti*, as identified through morphometric observations and PCR analysis [17]. Table 4 reveals that nematodes, such as *Capillaria* spp. are dominant across all three locations, demonstrating their adaptability to various environmental conditions. A previous study in Asia such as India, Pakistan, Nepal, Bhutan, Bangladesh, Sri Lanka, and the Maldives also found nematodes to be dominant in poultry, including ducks [18]. Environmental conditions, particularly humidity, influence the distribution of parasites. Research conducted in a zoo in China showed that high humidity increases the prevalence of nematodes, such as *Capillaria* spp., in various bird species such as Emu (*Dromaius novaehollandiae*) and Black crowned-crane (*Balearica pavonina*) [13]. These findings highlight the importance of implementing prevention and control strategies, such as maintaining good sanitation and regularly administering anti-parasitic medications. Proper deworming has been shown to effectively reduce parasite prevalence in poultry [19].

### Seasonal occurrence of helminths in ducks

Findings from this study agree with the previous study by Sumboh *et al*. [20]. Specific geographical locations, temperature, and humidity facilitate the growth and development of helminth larvae in the soil, with warm and humid conditions creating optimal environments for egg survival and transmission. Higher humidity during the rainy season may enhance the survival and transmission of nematodes and other parasites that thrive in wet conditions [21]. Nematode infections comprised a significant portion of all infections, with notable species such as *Capillaria* spp. and *Trichostrongylus* spp.

*Capillaria* spp. was the most prevalent species during the rainy season, detected in 15/64 (23.44%) ducks and had a slightly lower occurrence in the dry season, found in 31/137 (22.63%) ducks. However, thedifference between seasons was not statistically significant (p = 0.529). This relative stability suggests that the parasite is a persistent environmental reservoir, highlighting the need for ongoing monitoring [22, 23]. The absence of certain nematodes during the dry season, such as *Syngamus* spp. and *A. galli*, indicates that these species may have specific ecological requirements that are not met during dry conditions [24]. Conversely, the emergence of *Echinuria* spp. in the dry season (9.49%) suggests changes in the composition of helminth species populations, which may affect host immunity and health [25]. Cestode infections were relatively low, with only *Railletina* spp. being observed infrequently. The presence of protozoan parasites, particularly coccidia (18.75%), during the rainy season raises concerns about potential coinfections, which could exacerbate the overall health challenges faced by poultry populations [26, 27]. Such co-infections can lead to increased morbidity and mortality, negatively affecting the productivity and overall health of poultry [24].

The observed seasonal patterns emphasize the importance of tailoring helminth control strategies to align with seasonal dynamics. Strategies such as scheduled deworming during the rainy season and improved waste management help to reduce infection risks. During the rainy season, proactive measures, such as deworming and environmental management, should be prioritized to mitigate the higher risk of infection [21, 26].

Public, animal, and environmental health focus on overall well-being, not just disease prevention. Livestock management success is influenced by local farmers’ demographics, which affect technology adoption and business practices. Individual traits, including motives and values, also impact behavior and environmental interactions. Farmer education, both technically and managerially, significantly impacts the success of livestock businesses [28, 29]. Educating farmers about the signs of helminthiasis and the necessity of regular monitoring, especially during periods of increased rainfall, is crucial for maintaining poultry health [30]. Strategies for farmers to control parasitic diseases include limiting livestock access to frequently visited water sources, implementing good hygiene practices, monitoring high-risk diseases, and adapting strategies based on advancements in knowledge and disease dynamics [31].

Zoonotic parasitic infections in ducks primarily involve protozoa, such as *Giardia* spp., and the coccidia group, including *Eimeria* spp., with occurrence of 3% and 14%, respectively. Previous studies by Elmberg *et al*. [31], and Talazadeh *et al*. [32] indicate that wild geese can carry parasites such as *Cryptosporidium*, *Giardia*, and *Microsporidia*, but their role in direct transmission to humans or livestock is considered negligible and more linked to contaminated environments. Previous studies by Elmberg *et al*. [31] and Talazadeh *et al*. [32] underscore the importance of monitoring, prevention, and control of subclinical infections, particularly those with direct implications for public health. Evaluating the effectiveness of targeted deworming programs and alternative parasite control strategies could be achieved by using herbal anthelmintics based on local plants [33, 34].

Observations indicate that the use of active plant compounds has a significant effect on addressing livestock health issues and supports the mission of combating resistance and residues from chemical drugs [35–38].

## CONCLUSION

This study provides comprehensive insights into the occurrence and distribution of gastrointestinal parasites in local ducks raised at different altitudes in Yogyakarta, Indonesia. The findings indicate that 51.24% of the ducks examined were infected, with nematodes being the most predominant parasites, particularly *Capillaria* spp. (43.78%) and *Trichostrongylus* spp. (22.89%). Protozoa (coccidia) and cestodes (*Raillietina* spp.) were detected at lower frequencies. The occurrence of parasitic infections varied significantly with altitude, with higher infection rates in lowland (61.43%) and highland (61.54%) regions compared to coastal areas (30.30%). Seasonal variation also played a crucial role, as infection rates were significantly higher in the rainy season (67.19%) than in the dry season (43.80%), highlighting the influence of environmental conditions on parasite survival and transmission.

This study’s strength lies in its broad geographic coverage, allowing for a comprehensive assessment of altitude-related variation in parasite occurrence. The inclusion of both rainy and dry seasons provides valuable insights into the seasonal dynamics of infections. In addition, the study reflects real-world conditions of free-range duck farming, making the findings directly applicable to local farming practices. However, certain limitations should be acknowledged. The reliance on a single diagnostic method may have resulted in the underestimation of some parasite species, and the lack of molecular identification limits precise species differentiation. Furthermore, host-related factors such as age, diet, and immune status were not considered, which could influence susceptibility to infections.

Future research should focus on molecular characterization of parasites for more precise identification and potential zoonotic implications. Longitudinal studies covering multiple seasons and years would provide deeper insights into long-term trends. Further investigations are also needed to assess the impact of parasitic infections on duck productivity, including growth rates and egg production. Evaluating the effectiveness of targeted deworming programs and alternative parasite control strategies, such as herbal anthelmintics and improved farm biosecurity measures, would be beneficial for sustainable parasite management.

The findings of this study highlight the critical role of environmental factors in shaping parasite occurrence in local duck populations. Implementing targeted deworming programs, improving farm sanitation, and educating farmers on parasite monitoring can significantly reduce infection rates and enhance duck health and productivity. Future research should explore advanced diagnostic techniques, host-parasite interactions, and sustainable control measures to improve poultry health management in free-range farming systems.

## AUTHORS’ CONTRIBUTIONS

SA and IW: Conceptualized and designed the study. SA, IW, ZAB, and RM: Sample collection and laboratory analysis. AS, WW, FE, IF, SS, MS, HH, RZA, and ER: Data analysis and interpretation and manuscript preparation. SA, IW, IF, and ZAB: Drafted the manuscript. All authors actively participated in revision of the manuscript, provided intellectual input, and approved the final version for submission.
